# Influence of Internet Use on Commercial Health Insurance of Chinese Residents

**DOI:** 10.3389/fpubh.2022.907124

**Published:** 2022-07-08

**Authors:** Bao-Chang Xu, Xiao-Ni Xu, Jin-Chun Zhao, Meng Zhang

**Affiliations:** ^1^Department of International Economy and Trade, School of Economics, Qingdao University, Qingdao, China; ^2^Institution of Rural Development, Jiangsu Provincial Academy of Social Sciences, Nanjing, China; ^3^Nanjing Institute of Agricultural Mechanization, Ministry of Agriculture and Rural Affairs, Nanjing, China

**Keywords:** commercial health insurance, Internet use, binary probit, CGSS2017, China

## Abstract

As a necessary supplement to social medical insurance, commercial health insurance is an important part of the Healthy China strategy. This study, based on the Chinese General Social Survey (CGSS) data in 2017, uses the probit model to analyze and study the internal relationship between Internet use and commercial health insurance purchase of urban and rural residents. The research results show that the use of the Internet significantly promoted commercial health insurance purchases of residents, and the promotion effect for rural residents is apparently better than that among urban residents. In addition, the social level of residents is improved through the use of Internet, which can promote commercial health insurance purchases. It provides a significant reference value for the effective integration of Internet use and commercial health insurance, and the high-quality development of the modern insurance industry.

## Introduction

Commercial health insurance plays an important role in promoting economic development and maintaining social stability and establishing and improving the multi-level medical security system, as well as improving the health status of residents (1, 2), which is an indispensable part of the national medical security system. Lots of policies were successively introduced by the Chinese government to encourage and promote the development of commercial health insurance. In line with this, the State Council mentioned that it is required to accelerate the development of commercial health insurance and form a joint force with social medical insurance to meet the diversified health security needs of the masses in 2014. In addition, the State Council also mentioned that as of 2030, the modern commercial health insurance service industry should be further developed, as well as the proportion of commercial health insurance compensation expenditure in total health expenditure should be increased significantly. Moreover, in 2020, the regulatory authorities stressed the regulations about health insurance products and services should be improved, and the scale of the commercial health insurance market should exceed 2 trillion yuan by 2025. At present, commercial health insurance has become an important part of the multi-level protection system and has achieved remarkable results. In 2015, 9.93 percent of the industry-wide premium income is commercial health premium income. In 2020, the premium income of commercial health insurance reached 817.3 billion yuan, accounting for 17.11% of the whole industry's premium income. Although the overall growth rate of commercial health insurance is fast, there is still much room for improvement.

With the application and popularization of the Internet, the integration of commercial health insurance and the Internet has been continuously strengthened. At the initial stage of integration, the Internet was only used by insurance companies to brand promotion, business consultation, and product introductions, and almost no online transactions. Recently, with the continuous increase of Internet penetration and the expansion of the scale of netizens, commercial insurance companies regard the Internet as a new type of sales channel to gradually develop online services, such as product sales, policy inquiry, and claim settlement. By 2020, there are 146 insurance companies in China that have handled the Internet insurance business, and the premium income of the Internet has reached 79.795 billion yuan. At the same time, the two-way interactive platform is built by the Internet to strengthen the relationship between commercial insurance companies and consumers. Information sharing and portable communication are conducive to consumers to have a more comprehensive and in-depth understanding of insurance products, and improve financial knowledge to a certain degree, which is beneficial to increasing their insurance awareness and insurance needs, and affects commercial insurance purchase decisions of residents.

Therefore, considering the effective integration of the Internet and commercial health insurance in China, and traditional social insurance no longer meet the needs of risk prevention of residents, this study will explore the impact of Internet use on Chinese commercial health insurance purchase of residents and study its internal mechanism.

## Literature Review and Research Hypothesis

Regarding the research on residents purchasing commercial health insurance, many scholars at home and abroad conducted research from individual, family, and environmental aspects. In terms of individual factors, Showers et al. (3) found that there is a positive relationship between the age of residents and the demand for commercial insurance. However, the conclusions of gender on the demand for commercial insurance are inconsistent. According to a study in Taiwan, Lin et al. (4) found that the proportion of women purchasing insurance is higher than that of men. Luciano et al. (5) came to the opposite conclusion on the basis of a survey of Italian households. Pu et al. (6) argued that the higher the individual's education level, the higher the probability of purchasing insurance. Insurance knowledge is important for insurance purchase decisions (7). Lin et al. (4) constructed a financial literacy index and pointed out that consumers with a higher level of financial literacy are more likely to purchase life insurance. Kunreuther et al. (8) believed that if consumers lack relevant knowledge, they need to spend a lot of time and energy searching for insurance information, which will inhibit their demand for purchasing insurance, confirming the positive correlation between financial knowledge and, again, insurance purchase. In terms of family condition, Hammond et al. (9) found that family income has a wealth threshold effect on insurance purchases, that is to say, only when the family income is sufficient to maintain the basic standard of living, the family will be able to purchase insurance. The study of Albouy and Blagoutine (10) showed that the expansion of family size increases the demand for family insurance. The family demographic structure also affects insurance needs. Beck et al. (11) believed that the dependency ratio of young people (under 15 years of age) has no significant effect on life insurance consumption, while the dependency ratio of the elderly (over 64 years of age) has a greater impact on life insurance demand. Li et al. (12) believe that the youth dependency ratio has a positive impact on the demand for household commercial insurance, while the old-age dependency ratio has a negative impact on this demand. In terms of environmental factors, the Chinese government encourages farmers to participate in insurance to mitigate the impact of natural risks and market risks (13). Wang et al. (14) found that if the air pollution level is high, policyholders prioritize obtaining insurance for their children. Wang et al. (15), based on the data from the 2017 China Household Financial Survey (CHFS), the results showed that the rise in SO_2_ emissions can increase the tendency of residents to participate in commercial health insurance. Residents in eastern parts of China, people with high income, and female are more likely to purchase commercial health insurance facing SO_2_ pollution.

As to the impact of Internet use on insurance, most literature focuses on the influence of Internet on insurance industry. Salatin et al. (16) used fixed effects panel and GMM method to conduct empirical analysis, the regression results showed that the development of the insurance industry can be promoted by the improvement of information and communication technology. Naicker and Van Merve (17) found that mobile technology can improve the ability to perceive risks in the life insurance industry. Using computer-based information systems, Gavrilov et al. (18) argued that the Internet used in Health Insurance Funds would have a positive impact on policyholders, medical service providers, and companies when fulfilling their obligations and exercising their rights. On the basis of the New Zealand insurance industry, Yao (19) came to the conclusion that the Internet is used to promote products by most insurance companies, but few companies provide online transactions, so there is still considerable room for the development of website functions.

There were also some studies on the internal influencing mechanism between Internet use and the insurance industry. Based on American life insurance data, Brown et al. (20) found that the price of insurance products can be lowered by reducing the online search costs of consumers. Using data from the India insurance industry, Garven (21) argued that the consumers compare prices and product attributes between different insurance companies by using the Internet and reduce the information asymmetry between policyholders and insurance companies, which will further increase the number of insurance companies online services.

In conclusion, existing literature focused on the impact of Internet use on the insurance industry, while there is few research from a micro perspective, paying attention to the relationship between insurance purchases of residents and the use of the Internet. Therefore, this study analyzes the internal relationship between Internet use and commercial health insurance purchase of urban and rural residents based on the 2017 CGSS data, in order to provide a useful reference for the further integration of China's Internet and commercial health insurance and promote the high-quality development of China's commercial health insurance.

## Data Sources and Specification of Variables

### Data Sources

This paper uses data from CGSS in 2017. CGSS is a comprehensive and continuous academic survey project in China and is the main data source to study Chinese society. In this survey, data were comprehensively and systematically collected from multiple levels such as society, community, family, and individual, providing a nationwide and authoritative source of data samples for conducting scientific research and solving practical problems. There were a total of 12,582 valid questionnaires completed in 2017 CGSS. After filtering and processing data, 10,900 valid samples can be obtained.

### Variables Selection

In this paper, the explained variable is the commercial health insurance purchase of residents. There were 10 valid samples in the data of CGSS2017, among which 9,651 people did not purchase commercial health insurance, while 1,249 did.

The core explanatory variable used in this paper is the use of the Internet. This variable was obtained from question A28 in the CGSS questionnaire, “In the past year, your frequency of Internet use (including mobile Internet) is?.” The available answers are “Never,” “Rarely,” “Sometimes,” “Frequent,” and “Very Frequent.” In our study, the variable of Internet use is set to 0 if the answer is “Never” and other answers are set to 1.

The control variables of this paper are selected from two dimensions including individual and family. The detailed control variables are shown in Table 1.

**Table 1:** Variable definition and assignment.

**Variable**	**Variable assignment**
Internetuse	Yes = 1,No = 0
Insurance	Yes = 1, No = 0
Age	Age (years)
Age_2	Age squared
Gender	Male = 1, Female = 0
Edu	Whether it's high school/technical secondary school or above: Yes = 1, No = 0
Household	Town = 1, Country = 0
Marriage	Yes = 1, No = 0
Health	From 1 to 5, get better
ln_fincome	Take the logarithm of last year's household income
Familysize	Number of people living together currently

The explained variable commercial health insurance purchase of residents is a binary dummy variable. As seen in Table 2, the average value of commercial health insurance purchase of residents is 0.11, meaning that most people did not purchase commercial health insurance, and the awareness of participating in commercial health insurance needs to be improved. The average value of Internet use is 0.59, meaning that a large number of people use the Internet. The Internet can be further popularized to facilitate people's lives.

**Table 2:** Descriptive statistics of variables.

**Variable**	**Observation**	**Mean**	**Standard deviation**	**Minimum**	**Maximum**
Internetuse	10,900	0.59	0.49	0	1
Insurance	10,900	0.11	0.32	0	1
Age	10,900	50.84	16.37	19	85
Age_2	10,900	2852.78	1683.62	361	7,225
Gender	10,900	0.48	0.50	0	1
Edu	10,900	0.36	0.48	0	1
Household	10,900	0.64	0.48	0	1
Marriage	10,900	0.77	0.42	0	1
Health	10,900	3.48	1.09	1	5
ln_fincome	10,900	10.63	1.24	5.19	16.12
Familysize	10,900	2.80	1.37	1	12

## Model Selection and Empirical Result

### Model Selection

Since the explained variable is a binary dummy variable in the data of CGSS2017, the commercial health insurance purchase of residents is divided into 0 and 1. Zero (0) represents residents who do not purchase commercial health insurance and 1 is quite the opposite, representing residents who purchase commercial health insurance. Therefore, probit regression model is adopted to conduct empirical research and analysis.

The empirical regression model is set in the paper as below:


(1) $$\begin{array}{l} prob(insurance=1)=\beta_{0}+\beta_{1}Internetuse+\beta_{2}Age\\ +\beta_{3}Age\_2+\beta_{4}Gender+\beta_{5}Edu+\beta_{6}Marriage\\ +\beta_{7}Household+\beta_{8}\ln\;\_fincome+\beta_{9}Familysize+\mu \end{array}$$

Insurance represents whether residents purchase commercial health insurance, and Internet use represents whether residents are using the Internet. The other variables are selected to control the influence of possible factors on the regression results.

### Empirical Result

This study investigates the influence of Internet use on commercial health insurance purchases of urban and rural residents. All regressions are conducted based on the probit regression model. The regression result is shown in Table 3.

**Table 3:** Benchmark regression.

**Variable**	**(1)**	**(2)**
Internetuse	0.9046***	0.2473***
	(0.0408)	(0.0526)
Age		0.0406***
		(0.0080)
Age_2		−0.0005***
		(0.0001)
Gender		−0.0122
		(0.0349)
Edu		0.3800***
		(0.0403)
Household		0.1861***
		(0.0479)
Marriage		−0.0665
		(0.0495)
Health		0.0361*
		(0.0191)
ln_fincome		0.2621***
		(0.0214)
Familysize		−0.0246*
		(0.0144)
_N_	10,900	10,900
*R* ^2^	0.0767	0.1540

The ***, **, and * in the table, respectively show the significance under the significance level of 1, 5, and 10%, and the robust standard errors are shown in the brackets. The same as in the following tables.

As seen from the models (1) from Table 3, the coefficient obtained by regression when only adding the core explained variable is significant with a positive sign, meaning that there is a positive relationship between Internet use and residents' commercial health insurance purchase, that is to say, Internet use can significantly promote residents to purchase commercial health insurance. In model (2), a series of control variables at the individual and family level is added, and the coefficient of core explained variables obtained by regression is still significantly positive, which again confirms that Internet use has a positive influence on commercial health insurance purchase of residents.

In terms of control variables, as seen from the regression result, the coefficient of variable age is significantly positive, while the coefficient of age squared is significantly negative, meaning that there is an inverted U-shaped relationship between age and commercial health insurance purchase. That is to say, middle-aged people are more willing to purchase commercial health insurance. Compared with middle-aged people, the risk awareness of young people is relatively weak, and the health status of the elderly is poor, so higher premiums need to be charged by insurance companies. The coefficient of education level has also passed significance testing, meaning that residents with higher educational levels have rich financial knowledge that enables them to participate in commercial health insurance. The coefficient of variable household registration status is significantly positive, showing that urban residents pay more attention to commercial health insurance than rural residents, which also explains that urban residents have more awareness of risk prevention than that rural residents, intentionally purchasing commercial health insurance to prevent risk. The increase of family income can encourage residents to purchase commercial health insurance. The regression result of health shows that residents with better health status will purchase insurance. Generally, residents with poor health status put insurance companies at greater risk, so higher premiums will be charged by insurance companies.The regression result of family size is significant but negative, which means that the increase in number of households will weaken the demand for commercial health insurance.

### Robustness Test

To test the stability of the explanation of evaluation results by evaluation methods and indicators, it is necessary to conduct a robustness test. This paper conducts a robustness test using the method of substituting the independent variable.

#### Substituting Independent Variable

This paper chooses to replace the measurement method of the explanatory variable to conduct robustness test. The core variable of Internet use obtained from question A29 in the CGSS questionnaire is: “Among the above media, which one is your main source of information?.” The available answers are “Newspapers,” “Magazine,” “Broadcast,” “Television,” “Internet (including mobile Internet),” and “Mobile phone customized message.” The variable the use of the Internet is set to 1 if the answer is “Internet (including mobile Internet),” and other answers are set to 0.

As can be seen from Table 4, the coefficient of Internet use is statistically significant at the 1% significance level with a positive sign. This indicates that the positive relationship between the use of the Internet and commercial health insurance purchases does not change, which means that the regression results of all samples have good robustness. After adding a series of control variables at the individual and family levels, the significance and regression coefficients of each control variable have not changed much. In conclusion, regression results based on all samples are robust.

**Table 4:** Robustness test.

**Variable**	**(1)**	**(2)**
Internetuse	0.7869***	0.2628***
	(0.0335)	(0.0460)
Age		0.0470***
		(0.0080)
Age_2		−0.0005***
		(0.0001)
Gender		−0.0133
		(0.0350)
Edu		0.3756***
		(0.0403)
Household		0.1900***
		(0.0476)
Marriage		−0.0739
		(0.0493)
Health		0.0387**
		(0.0191)
ln_fincome		0.2617***
		(0.0213)
Familysize		−0.0233
		(0.0144)
N	10,858	10,858
*R* ^2^	0.0753	0.1560

** means the coefficient is significant at the 5% level. *** means the coefficient is significant at the 1% level.

## Further Discussion

### Heterogeneity Test

As can be seen from Table 5, there are 3,911 urban samples and 6,989 rural samples. In the regression results of urban and rural samples, the regression results of Internet use and the core explanatory variable, have passed all the significant testing, and the regression coefficients are positive. This means that, for urban and rural residents, the use of In the Internet positively affects commercial health insurance purchases. In addition, the result of significant testing shows that the data for rural areas are better than that of the urban areas, meaning that Internet use of rural residents has a greater influence on commercial health insurance purchases than that of urban residents.

**Table 5:** Heterogeneity test-Urban-rural differences.

**Variable**	**Rural area**		**Urban area**	
	**(1)**	**(2)**	**(3)**	**(4)**
Internetuse	0.6346***	0.2729***	0.8565***	0.2546***
	(0.0738)	(0.0972)	(0.0529)	(0.0653)
Age		0.0279		0.0452***
		(0.0171)		(0.0092)
Age_2		−0.0003*		−0.0005***
		(0.0002)		(0.0001)
Gender		0.1571**		−0.0636
		(0.0775)		(0.0394)
Edu		0.4214***		0.3581***
		(0.0957)		(0.0443)
Marriage		0.0033		−0.0750
		(0.1055)		(0.0569)
Health		0.0163		0.0482**
		(0.0375)		(0.0224)
ln_fincome		0.2129***		0.2820***
		(0.0445)		(0.0244)
Familysize		0.0406		−0.0490***
		(0.0257)		(0.0173)
N	3,899	3,899	6,959	6,959
*R* ^2^	0.0545	0.1132	0.0534	0.1205

** means the coefficient is significant at the 5% level. *** means the coefficient is significant at the 1% level.

Therefore, it can be concluded that there is a positive correlation between the use of Internet and commercial health insurance purchase of urban and rural residents, and there is heterogeneity among rural and urban samples, wherein the correlation is more significant among rural samples.

### Mediation Effect Test

In order to study the detailed influence mechanism of Internet use on commercial health insurance purchase, we adopt a step-test regression coefficient method to examine how Internet use affects commercial health insurance purchase.

In this test, the independent variable is Internet use, the dependent variable is commercial health insurance purchase of residents, and the mediating variable is social level of residents, which is from question A311: “In the past year, do you often socialize or visit in your free time?.” The available answers are “Never,” “Rarely,” “Sometimes,” “Frequent,” and “Very Frequent,” and assigned values from 1 to 5, with 1 being “Never” and 5 being “Very Frequent.” To summarize, the larger the number, the higher the social level of residents. This study will conduct research on whether Internet use can affect commercial health insurance purchases by influencing the social level of residents.


(2) $$\begin{array}{r} Y=cX+e_{1} \end{array}$$

(3) $$\begin{array}{r} M=aX+e_{2} \end{array}$$

(4) $$\begin{array}{r} Y=c^{\prime }X+bM+e_{3} \end{array}$$

The method of step-test regression coefficient is commonly used to test the mediation effect. This method includes three steps, and the first step is to analyze the total effect of X to Y, meaning to test the significance of regression coefficient c in equation (3), specifically, which refers to in this paper as the total effect of Internet use on commercial health insurance purchase of residents. The regression result is shown in Table 6.

**Table 6:** The relationship between residents' health and commercial insurance.

**Insurance**	**Coef**	**Std.err**	***z***	***p* > |z|**	**[95% Conf. Interval]**	
Internetuse	0.2504447	0.0528319	4.74	0.000	0.1468961	0.3539932
_cons	−5.332939	0.3002147	−17.76	0.000	−5.921349	−4.744529

As can be seen from Table 6, coefficient c is significant and the regression result is 0.250, meaning that the use of Internet can significantly promote residents to participate in commercial health insurance, and then, we can proceed to the second step.

The second step is to test the correlation between independent variable X and mediating variable M, that is to say, testing the significance of coefficient a in equation (4). It refers to the influence of Internet use on the social level of residents. The regression result is shown in Table 7.

**Table 7:** The relationship between insurance participation and interact.

**Social level**	**Coef**	**Std.err**	***z***	***p* > |z|**	**[95% Conf. Interval]**	
Internetuse	0.1911618	0.0459592	4.16	0.000	0.1010835	0.2812402
_cons	0.6616746	0.2345751	2.82	0.005	0.2019159	1.121433

It can be seen that coefficient a is significant and the sign is positive. The regression result shows that the use of the Internet significantly improves the social level of residents, and the coefficient a is 0.191. The third step can be taken.

In the third step, we add the social level of residents, which is the mediating variable to the model, to test the significance of coefficients c' and b in equation (5), and to analyze the relationship between Internet use and participation in commercial insurance of residents. The regression results are shown in Table 8.

**Table 8:** Total effect.

**Insurance**	**Coef**	**Std.err**	***z***	***p* > |z|**	**[95% Conf. Interval]**	
Social level	0.1825944	0.0619912	2.95	0.003	0.0610939	0.3040948
Internetuse	0.2475008	0.0529434	4.67	0.000	0.1437335	0.351268
_cons	−5.476637	0.3011626	−18.18	0.000	−6.066905	−4.886369

It can be seen that coefficients c' is 0.248 and b is 0.183, and they are both significantly positive, meaning that adding the social level of residents doesn't change the positive relationship between Internet use and commercial insurance purchase of residents. There is also a positive relationship between the social level of residents and residents' commercial health insurance purchase. These results show that the social level has played the mediating role between the use of the Internet and commercial health insurance purchases.

The complete explanation of result of step-test regression coefficient method is as below: the total effect of Internet use on commercial health insurance purchase is 0.250, which is significant, and the direct effect of Internet use on commercial health insurance purchase is 0.248, which is significant, and the influence is obvious. The mediating effect of Internet use in promoting residents' commercial health insurance purchases by improving their social level is 0.183, and it accounted for 73.20% of the total effect.

## Conclusion and Inspiration

This study uses data from CGSS2017 to research influence of Internet use on commercial health insurance purchases of urban and rural residents. The result shows that: (1) Internet use significantly promotes residents purchase commercial health insurance; (2) For urban areas and rural areas, the influence of Internet use on commercial health insurance purchase is different. Specifically, the level of influence on rural residents is higher than that of urban residents, that is to say, Internet use has a more positive influence on commercial health insurance purchases for rural residents; (3) In terms of influence mechanism, Internet use can promote commercial insurance purchase by increasing social level of residents, meaning that social level of residents played the part of the mediating effect in the influence of Internet use on commercial health insurance purchase of residents.

In view of the above conclusion and the actual situation in China, the following enlightenment is obtained in this study: First, the construction of Internet infrastructure should be further increased by the government in order to give full play to the role of the Internet in promoting commercial insurance purchase of urban and rural residents. Second, the Internet platform should be fully used by commercial insurance companies, and the information transmission function of the Internet can be used to improve insurance awareness of residents, especially those who are not sociable. Third, relevant regulations and laws should be formulated by relevant departments to guide and regulate the development of Internet insurance. As to insurance consumers, legal awareness should be further improved, and seek legal help when their legitimate rights are infringed.

## Data Availability Statement

The original contributions presented in the study are included in the article/supplementary material, further inquiries can be directed to the corresponding author.

## Author Contributions

B-CX: conceptualization, methodology, and software. X-NX: data curation and writing. J-CZ: methodology and writing. MZ: visualization and investigation. All authors contributed to the article and approved the submitted version.

## Funding

This research is supported by the National Natural Science Foundation of China (71903105 and 71803101).

## Conflict of Interest

The authors declare that the research was conducted in the absence of any commercial or financial relationships that could be construed as a potential conflict of interest.

## Publisher's Note

All claims expressed in this article are solely those of the authors and do not necessarily represent those of their affiliated organizations, or those of the publisher, the editors and the reviewers. Any product that may be evaluated in this article, or claim that may be made by its manufacturer, is not guaranteed or endorsed by the publisher.
